# Supplementary material for: Smooth Interpolating Curves with Local Control and Monotone Alternating Curvature
Source: Comput Graph Forum. 2022 Oct 6;41(5):25–38. doi: 10.1111/cgf.14600 (PMC9827861; doi:10.1111/cgf.14600)
Supplement: Supplementary file 1 — Supplement Material [file CGF-41-25-s001.zip › Local-Smooth-Interpolating-MonoCurvature/extern/clothoids/docs/api-matlab/function_a00062_1a1ab9fbe6bf4f16efdaef918d8d6e1bc8.html]

Function TriTriOverlap — Clothoids v2.0.9

### Navigation

- index
- toc
- previous
- Clothoids »
- MATLAB API »
- Function TriTriOverlap

# Function TriTriOverlap¶

- Defined in File TriTriOverlap.m

## Function Documentation¶

function TriTriOverlap( p0, p1, p2, q0, q1, q2)¶
:   TriTriOverlap: Check if two triangles overlap

    **Usage:**

    ```
    intersect = TriTriOverlap( p0, p1, p2, q0, q1, q2 );
    ```

    **On input:**

    - p0, p1, p2 = coordinates of first triangle
    - q0, q1, q2 = coordinates of second triangle

    **On output:**

    - intersect = true if the triangle overlap

### Quick search

### Table of Contents

- Matlab Interface Manual
- C++ API
- MATLAB API

«
hide menu

menu
sidebar
»

### Navigation

- index
- toc
- previous
- Clothoids »
- MATLAB API »
- Function TriTriOverlap

© Copyright 2021, Enrico Bertolazzi and Marco Frego.
Created using Sphinx 4.2.0.
